# Longitudinal associations of lifestyle factors and weight status with insulin resistance (HOMA-IR) in preadolescent children: the large prospective cohort study IDEFICS

**DOI:** 10.1186/s12966-016-0424-4

**Published:** 2016-09-02

**Authors:** Jenny Peplies, Claudia Börnhorst, Kathrin Günther, Arno Fraterman, Paola Russo, Toomas Veidebaum, Michael Tornaritis, Stefaan De Henauw, Staffan Marild, Dénes Molnar, Luis A. Moreno, Wolfgang Ahrens, on behalf of the IDEFICS consortium

**Affiliations:** 1 Leibniz Institute for Prevention Research and Epidemiology – BIPS, Bremen, Germany; 2 Faculty of Human and Health Sciences, Institute for Public Health and Nursing Research, Bremen University, Bremen, Germany; 3 MVZ Dortmund Dr. Eberhard und Partner, Dortmund, Germany; 4 Epidemiology & Population Genetics, Institute of Food Sciences, CNR, Avellino, Italy; 5 National Institute for Health Development, Tallinn, Estonia; 6 Research & Education Institute of Child Health, Strovolos, Cyprus; 7 Department of Public Health, Ghent University, Ghent, Belgium; 8 Department of Public Health and Community Medicine, The Queen Silvia Children’s Hospital, Göteborg, Sweden; 9 National Institute of Health Promotion, University of Pécs, Gyermekklinika, Pécs, Hungary; 10 GENUD (Growth, Exercise, Nutrition and Development) Research Group, School of Health Sciences, University of Zaragoza, Zaragoza, Spain; 11 Epidemiological Methods and Etiologic Research, Leibniz Institute for Prevention Research and Epidemiology – BIPS, Achterstraße 30, 28359 Bremen, Germany

**Keywords:** Insulin resistance, HOMA-IR, Physical activity, Accelerometer, Obesity, Pre-pubertal children, Cohort study

## Abstract

**Background:**

This study investigates prospective associations of anthropometrical and lifestyle indices with insulin resistance (IR) in European children from the IDEFICS cohort. Insulin resistance (IR) is a growing concern in childhood obesity and a central aspect of the metabolic syndrome (MS). It most likely represents the link between obesity and type 2 diabetes.

**Methods:**

This longitudinal study included 3348 preadolescent children aged 3 to 10.9 years from 8 European countries who were observed from 2007/2008 to 2009/2010. The main outcome measure in the present analysis is HOMA-IR (homeostasis model assessment as a common proxy indicator to quantify IR) at follow-up and in its longitudinal development. Anthropometrical measures and lifestyle indices, including objectively determined physical activity, were considered, among others factors, as determinants of IR. Prospective associations between IR at follow-up and anthropometrical and lifestyle indices were estimated by logistic regression models.

**Results:**

Country-specific prevalence rates of IR in the IDEFICS cohort of European children showed a positive trend with weight category. Prospective multivariate analyses showed the strongest positive associations of IR with BMI z-score (OR = 2.6 for unit change from the mean, 95 % CI 2.1–3.1) and z-score of waist circumference (OR = 2.2 for unit change from the mean, 95 % CI 1.9–2.6), which were analysed in separate models, but also for sex (OR = 2.2 for girls vs. boys, 95 % CI 1.5–3.1 up to OR 2.5, 95 % CI 1.8–3.6 depending on the model), audio-visual media time (OR = 1.2 for an additional hour per day, 95 % CI 1.0–1.4 in both models) and an inverse association of objectively determined physical activity (OR = 0.5 for 3^rd^ compared to 1^st^ quartile, 95 % CI 0.3–0.9 in both models). A longitudinal reduction of HOMA-IR was accompanied with a parallel decline in BMI.

**Conclusions:**

This study is, to our knowledge, the first prospective study on IR in a preadolescent children’s population. It supports the common hypothesis that overweight and obesity are the main determinants of IR. Our data also indicate that physical inactivity and a sedentary lifestyle are likewise associated with the development of IR, independent of weight status. The promotion of physical activity should thus be considered as an equal option to dietary intervention for the treatment of IR in the paediatric practice.

## Background

Insulin resistance (IR), a reduced physiological response of the peripheral tissues to normal levels of insulin, is a growing concern in childhood obesity, although not all obese people are insulin resistant and IR may also occur in nonobese children and adults [1]. IR is also a central aspect of the metabolic syndrome (MS) and most likely a link between obesity and type 2 diabetes [2–6]. Given the constantly growing prevalence of metabolic disorders in children and adolescents [7], it appears to be important to identify children at risk before clinical symptoms occur.

Several risk factors for IR have been suggested, e.g. weight status [8], measures of central and peripheral adiposity [9, 10], dietary factors such as intake of total fat or saturated fat [11, 12], physical inactivity [13], poor physical fitness [14], low or high birth weight for gestational age [7, 15], and maternal factors like gestational diabetes [16] or unbalanced maternal nutrition [17]. Nevertheless, in children, population-based epidemiological data on the determinants of IR are still rare and mostly available from cross-sectional studies. A recent review [18] on the clustering of obesogenic behaviours in children or adolescents concluded that further research is needed particularly in younger children and from longitudinal studies.

The prevalence of MS in children has recently been described in a systematic review [19] that related to IR as one of several possible criteria of MS. Estimation of prevalence in this review was not straightforward because many different criteria had been used to define MS in children. The median prevalence was overall 3.3 % (range 0–19.2 %), 11.9 % (range 2.8–29.3 %) in overweight children, and 29.2 % (range 10–66 %) in obese children, when studies in all ethnicities were considered. For European children only, prevalence tended to be slightly lower. Two new definitions of paediatric MS were also suggested by the IDEFICS (“Identification and prevention of dietary- and lifestyle-induced health effects in children and infants”) study group [20], one for monitoring purposes (with HOMA-IR ≥90th percentile amongst other criteria) and one to define a cut point for clinical action (with HOMA-IR ≥95th percentile amongst other criteria). When HOMA-IR alone is considered prevalence proportions are usually higher than those for MS [8, 21], but data from population based studies in children are still limited.

The importance of metabolic health in the presence of obesity has been of growing research interest in the last years. Metabolically healthy obesity is apparently associated with lower levels of adiposity in youth [22, 23] and a high level of physical activity (PA) in adults [22, 23]. Increased PA and cardiorespiratory fitness are also believed to attenuate the risk of cardiovascular disease, type 2 diabetes, and MS, independent of weight status [24]. Physical fitness and physical activity are closely related and have been shown to correlate well in children [25]. The Lancet Physical Activity Series working group estimated that worldwide in 2008, physical inactivity caused 6 % of the burden of disease from coronary heart disease and 7 % of type 2 diabetes, as well as 9 % of premature mortality [26]. A meta-analysis on the influence of cardiorespiratory fitness and weight status on mortality from all causes showed that overweight and obese fit individuals had similar mortality risks as normal weight fit individuals. Compared to normal weight fit individuals, unfit individuals had twice the risk of mortality regardless of their body mass index (BMI) [27].

The present study investigates the prospective associations between anthropometrical and lifestyle indices with IR in preadolescent European children. It also shows the development of HOMA-IR-values in relation to the longitudinal development of weight status between the two IDEFICS surveys.

## Methods

### Study design

IDEFICS is an Integrated Project within the 6th Framework Programme of the European Commission aimed at the investigation of diet- and lifestyle-related diseases and their prevention. The baseline survey was conducted in the school year 2007/2008 and included 16,228 pre-school and primary school children aged 2 to 9 years from eight European countries. The survey comprised anthropometrical measurements and examinations of children as well as parental self-completion questionnaires on lifestyle habits and dietary intakes of children. Biomarkers were analysed in blood, urine, and saliva samples. Standardised procedures were used by all survey centres. Venous blood was collected from 9185 of the IDEFICS children. The majority of children were re-examined after 2 years during a follow-up survey (*n* = 11,292 plus 2517 newly recruited children). The background of the study, its research goals and instruments have been described elsewhere in detail [28].

### Study sample

After exclusion of non-fasting children, children with diagnosed diabetes, children who had missing data for waist circumference or one of the laboratory analyses of interest, and children from Cyprus (due to the very small sample size that fulfilled the requirements), 6970 and 6708 children remained in the data sets for baseline and follow-up survey, respectively. The present analyses are based on the 3348 children who were part of both groups. For the prospective analyses, also children with IR at baseline (HOMA-IR ≥95th percentile, according to Peplies et al. [29]) were excluded, which further reduced the sample to 3125 children. Due to missing information for some of the potential risk factors or confounders, some analyses were conducted in smaller samples (numbers are indicated in the tables). A selection effect could be excluded as characteristics of subgroups (children with data on sleep duration or children with accelerometer data) only differed by the first or second decimal compared to the whole study sample (data not shown).

### Physical examination

The physical examination programme during the two IDEFICS surveys covered standard anthropometric measures, i.e. height (SECA 225), weight (TANITA BC 420 SMA), and circumferences of waist, hip, upper arm, and neck (SECA 200), as well as skinfold thicknesses (subscapular and triceps) (Holtain skinfold caliper), foot-to-foot bioelectrical impedance (TANITA BC 420 SMA), and the measurement of blood pressure and pulse rate (Welch Allyn 4200B) [28]. BMI was calculated as weight (in kg) divided by height squared (in m). Sex-specific BMI categories were interpolated for continuous age according to the extended IOTF criteria [30].

### Sleep duration

Information on sleep duration was collected in the context of a standardised 24-h recall called SACINA (self-administered children and infants nutrition assessment). SACINA is a computer-based instrument filled out by the parents/guardians of participating children with the assistance of a study nurse. Next to questions on dietary intakes, parents were asked about their children’s get up and bed time (hour/minute) of the previous day. Sleep duration on weekday nights was calculated resulting in a continuous estimate of sleep hours per night as described previously [31, 32].

### Insulin resistance

Fasting insulin and HOMA-IR (homeostasis model assessment to quantify IR) have been suggested among others as surrogate markers for screening purposes in adults [33]. The gold standard method to measure insulin sensitivity, the hyperinsulinemic euglycemic clamp, is invasive and very labour- and time-intensive, and thus not feasible in epidemiological research. Estimates of IR from HOMA-IR have been shown to correlate well with estimates from the clamp-technique [34], especially in healthy populations [35]. IR was defined as HOMA-IR ≥95^th^ percentile, calculated for half year age groups [29].

### Blood samples

Children participating in the IDEFICS surveys were asked to donate fasting venous blood samples. If consent was not given for venous blood withdrawal, capillary blood was taken with the consent of the parents and children. For the present analyses, only children with venous blood samples were included. Pre-analytical processing of blood samples was done at the local survey centres or at local laboratories. Samples were frozen at −80 °C and shipped to a central laboratory (accredited according to ISO 15189) for later analysis. Details on the biological sampling procedures can be obtained from a previous publication [36]. Blood glucose was assessed on site at each study centre by point-of-care analysis using the Cholestech LDX analyser (Cholestech®, Hayward, CA, USA) either in venous or capillary blood. Insulin was determined by electrochemiluminescence immunoassay in the central laboratory. HOMA-IR was calculated as fasting insulin (μIU/ml) × fasting glucose (mg/dl)/405.

### Questionnaire data

Data on education, lifestyle habits and dietary intakes of children was retrieved from parental self-completion questionnaires. Parental education was used as a proxy of socioeconomic status. It was coded country-by-country according to the International Standard Classification of Education (ISCED) [37]. For the analyses, the maximum ISCED level of both parents was considered. Media use was used as a proxy of sedentary behavior. It was described by the time spent with audiovisual media (hours/week) and the number of audiovisual media devices (TV, Computer, Internet connection, DVD player, Playstation, Game console) located in the child’s bedroom. For dietary assessment, propensity scores for sugar and fat consumption of children were developed from a parental food frequency questionnaire [38, 39]. This questionnaire recorded the child’s consumption of certain obesity-promoting or -inhibiting food items from a given list of foods [40] on a typical week, excluding foods provided in school or day care setting. Based on the food frequency questionnaire, the estimation of energy intake or total food intake was thus not possible. A continuous index was developed, using the total weekly frequency for high-sugar or high-fat items divided by the individual’s total consumed food frequencies.

### Physical activity

Activity data (time-varying accelerations) of a subset of participating children was recorded by uniaxial accelerometers (ActiGraph® GT1M or Actitrainer, LLC, Pensacola, FL, USA). Accelerometers were mounted on the right hip with an elastic belt ensuring close contact with the body. Activity data was analysed on the basis of a 60 s epoch. Included children had at least three measurement days and a minimum of 8 h of valid accelerometer wear time per day. Non-wear time was assumed for 20 min of consecutive zero counts. Time spent performing either moderate or vigorous intensity of PA (average minutes over all valid recording days) was calculated according to the cut points proposed by Evenson [41]. A detailed description of IDEFICS accelerometer data can be found elsewhere [42].

### Quality assurance

All measurements followed detailed standard operation procedures (SOPs) which were pre-tested before the baseline survey [43]. Field personnel from each study centre participated in a central training course. Site visits were conducted at all study locations during field surveys to check adherence to the SOPs. Questionnaires were developed in English, translated to local languages, and checked for translation errors after back-translation. All technical equipment and laboratory materials were purchased centrally to maximise comparability of data. Laboratory analyses were conducted at the central laboratory which was accredited according to ISO 15189.

### Statistical analyses

Data of parameters with normal distribution are presented as mean values (± standard deviation); data of parameters with skewed distributions are presented as median (25th, 75th percentile). Normality of distribution was assessed by the Kolmogorov-Smirnov test. Prevalence of IR was calculated using the age- and sex-specific 95th percentile of HOMA-IR derived from normal weight IDEFICS children as cut off value for each half year age group [29]. Furthermore, an age- and sex-specific z-score was calculated for HOMA based on this healthy paediatric population. Delta z-IR was calculated as the difference between the z-scores of HOMA-IR-values at T1 and T0 to depict the development of HOMA-IR between the two surveys. Logistic regression analyses was limited to children without IR at T0, i.e. to all children with a HOMA-IR <95th percentile in their corresponding half year age groups (cut-off ranged from 1.5 to 2.9 for 3–9 year old girls and 1.3–2.7 for the respective boys) [29]. An indicator variable for presence of HOMA-IR at follow-up (HOMA-IR above or below 95^th^ percentile) was defined as dependent variable for the logistic regression analyses. Univariate logistic regression analyses were conducted for sex, age (continuous), ISCED-level, BMI, BMI z-score, z-score of waist circumference (continuous and in quartiles), audio-visual media time (in quartiles), number of media in bedroom (in quartiles), propensity scores for sugar and fat consumption (in quartiles), sleep duration (continuous), and for accelerometer data as time spent in moderate to vigorous physical activity (MVPA, in quartiles) both, for all children and children with normal weight only. Multivariate mixed logistic models were calculated separately for the exposures BMI z-score and z-score of waist circumference to avoid collinearity. Covariables were added to the model if they were significant in the univariate analysis for normal weight children, i.e. audio-visual media time (as continuous variable), sex, age, ISCED level and time spend in MVPA (in quartiles). A random country effect was included in the model to account for the clustered study design. ‘Number of media in bedroom’ was dropped in favour of audio-visual media time as both measure a similar construct, thus to avoid collinearity. Even though no significant effect was seen for the nutritional covariables in the univariate analyses, fat consumption score (as continuous marker) was included into the model due to the biological connection of the children’s nutrition and the biological markers. Odds ratios (ORs) and 95 % confidence limits were calculated for all children and for boys and girls separately. All analyses were performed using SAS® statistical software version 9.3 (SAS Institute, Inc., Cary, NC).

## Results

Characteristics of the study population at baseline (T0) and during the follow-up survey (T1) are presented in Table 1. Values for all anthropometrical measurements and biological markers of study participants were higher at T1. Highest parental ISCED level was about equal between the surveys and among the sexes. Girls and boys exhibited no differences as to age and anthropometrical measures. There were small sex differences for the biochemical markers: insulin values were higher in girls (12 % at T0 and 20 % at T1) and glucose levels were slightly higher in boys (3 %). Also media consumption, i.e. time spent with audiovisual media and number of media in bedroom, was higher in boys, and time spent in MVPA was 30 % higher in boys.

**Table 1:** Characteristics of study population

	Baseline survey T0			Follow-up survey T1		
	All	Boys	Girls	All	Boys	Girls
N	3348	1743	1605	3348	1743	1605
Age (years) ^a^	6.4 (± 1.7)	6.3 (± 1.7)	6.4 (± 1.6)	8.4 (± 1.7)	8.3 (± 1.7)	8.4 (± 1.6)
*Anthropometry*						
Weight (kg) ^a^	23.9 (± 6.9)	24.0 (± 6.9)	23.9 (± 6.9)	30.6 (± 9.1)	30.7 (± 9.1)	30.6 (± 9.1)
Height (cm) ^a^	120.0 (± 12.0)	120.1 (± 12.0)	119.9 (± 12.0)	132.3 (± 11.3)	132.4 (± 11.2)	132.3 (± 11.4)
BMI (kg/m^2^) ^a^	16.3 (± 2.4)	16.3 (± 2.3)	16.3 (± 2.4)	17.2 (± 3.0)	17.1 (± 3.0)	17.2 (± 3.0)
Waist circumference (cm) ^a^	54.7 (± 6.7)	55.0 (± 6.8)	54.4 (± 6.6)	59.1 (± 8.4)	59.4 (± 8.5)	58.9 (± 8.3)
*Biochemical markers*						
Fasting glucose (mmol/l) ^a^	4.6 (± 0.5)	4.7 (± 0.5)	4.6 (±0.5)	4.8 (± 0.5)	4.8 (± 0.5)	4.7 (± 0.5)
Fasting insulin (pmol/l) ^b^	25.2 (15.6, 38.4)	23.9 (14.4, 37.1)	26.7 (17.4, 39.7)	38.0 (26.1, 55.4)	35.1 (23.6, 50.6)	42.1 (28.7, 61.6)
HOMA-IR ^b^	0.7 (0.4, 1.2)	0.7 (0.4, 1.1)	0.8 (0.5, 1.2)	1.1 (0.8, 1.7)	1.1 (0.7, 1.6)	1.3 (0.8, 1.8)
*Other variables*						
Time spent with audio-visual media (h/day) ^b c^	1.5 (1.0, 2.2)	1.6 (1.0, 2.2)	1.4 (1.0, 2.0)	1.9 (1.2, 2.5)	1.9 (1.2, 2.8)	1.8 (1.0, 2.4)
Number of media in the child’s bedroom ^a c^	0.8 (± 1.2)	0.9 (± 1.3)	0.7 (± 1.2)	1.0 (± 1.4)	1.1 (± 1.4)	1.0 (± 1.4)
Fat consumption propensity score ^a c d^	25.5 (± 9.3)	25.4 (± 9.4)	25.5 (± 9.2)	not available	not available	not available
Sugar consumption propensity score ^a c d^	25.0 (± 11.3)	25.3 (± 11.5)	24.7 (± 11.2)	not available	not available	not available
Highest parental education (ISCED-level) ^a c^	4.0 (± 1.2)	4.0 (± 1.2)	3.9 (± 1.2)	4.0 (± 1.2)	4.0 (± 1.2)	4.0 (± 1.2)
N with data on sleep duration	1836	961	875	1450	763	687
Sleep duration on weekdays	10.2 (± 0.9)	10.2 (± 1.0)	10.1 (± 0.9)	9.8 (± 1.0)	9.9 (± 1.0)	9.8 (± 1.0)
N with accelerometer data	1967	1029	938	1793	920	873
Time spent in MVPA (average minutes/day) ^a e^	41.2 (± 21.2)	46.0 (± 22.8)	36.0 (± 18.0)	43.4 (± 22.6)	48.5 (± 24.6)	38.0 (± 19.0)

^a^Data are presented as mean (± standard deviation), ^b^ Data are presented as median (25^th^, 75^th^ percentile),

^c^Variables with missing data, descriptive statistics based on slightly smaller numbers of children (*N* ≥3055)

^d^Propensity to consume items high in fat or sugar resp., relative to frequency of all items on food frequency questionnaire

^e^ *MVPA* moderate to vigorous physical activity

Prevalence rates of IR at follow-up were determined for children in the different BMI categories and are shown in Table 2, stratified by country. IR prevalence clearly showed an increasing trend with BMI, from an overall 2.2 % among thin (underweight) children and 10.9 % in normal weight, via 26.5 % in overweight, reaching a remarkable 66.7 % in obese children. This trend can be seen, both, in countries with a high amount of overweight and obese children like Italy, and in countries with very low rates of overweight and obesity like Sweden.

**Table 2:** Prevalence of insulin resistance at follow-up by BMI categories according to Cole & Lobstein [30]

Country	Insulin resistance	Thin	Normal weight	Overweight	Obese	All
Italy	HOMA-IR ≥ p95^a^	1	27	44	64	136
		*8.3 %*	*13.9 %*	*35.5 %*	*68.0 %*	*32.1 %*
	All	12 (2*.8 %)*	194 (*45.8 %)*	124 (*29.3 %)*	94 (*22.2 %)*	424 (*100.0 %)*
Estonia	HOMA-IR ≥ p95^a^	3	35	18	11	67
		*7.1 %*	*11.9 %*	*40.9 %*	*84.6 %*	*17.0 %*
	All	42 (10.7 %)	295 (*74.9 %*)	44 (*11.2 %)*	13 (*3.3 %)*	394 (*100.0 %)*
Belgium	HOMA-IR ≥ p95^a^	2	11	11	5	29
		*4.2 %*	*3.7 %*	*42.3 %*	*55.6 %*	*7.6 %*
	All	48 (*12.5 %)*	300 (*78.3 %)*	26 (*6.8 %)*	9 (*2.4 %)*	383 (*100.0 %)*
Sweden	HOMA-IR ≥ p95^a^	2	30	19	5	56
		*3.8 %*	*7.7 %*	*36.5 %*	*62.5 %*	*11.1 %*
	All	52 (*10.3 %)*	391 (*77.7 %)*	52 (*10.3 %)*	8 (*1.6 %)*	503 (*100.0 %)*
Germany	HOMA-IR ≥ p95^a^	2	27	16	8	53
		*5.4 %*	*13.1 %*	*39.0 %*	*88.9 %*	*18.1 %*
	All	37 (*12.6 %)*	206 (*70.3 %)*	41 (14*.0 %)*	9 (*3.1 %)*	293 (*100.0 %)*
Hungary	HOMA-IR ≥ p95^a^	8	89	46	30	173
		*6.7 %*	*18.2 %*	*44.7 %*	*69.8 %*	*22.9 %*
	All	120 (*15.9 %)*	488 (*64.7 %)*	103 (*13.7 %)*	43 (*5.7 %)*	754 (*100.0 %)*
Spain	HOMA-IR ≥ p95^a^	0	31	33	17	81
		*0.0 %*	*7.5 %*	*27.1 %*	*50.0 %*	*13.6 %*
	All	28 (4*.7 %*)	413 (*69.2 %)*	122 (*20.4 %)*	34 (*5.7 %)*	597 (*100.0 %)*
All countries	HOMA-IR ≥ p95^a^	8	250	187	140	595
		*2.2 %*	*10.9 %*	*36.5 %*	*66.7 %*	*17.8 %*
	All	339 (*10.1 %)*	2287 (6*8.3 %)*	512 (*15.3 %)*	210 (*6.3 %)*	3348 (*100.0 %)*

^a^Age- and sex-specific 95^th^ percentiles (p95) from Peplies et al. [29]

Longitudinal data were analysed for time varying exposure of weight status on IR (Table 3). IR was considered as time-varying outcome (delta z IR), which was negative (a lower HOMA-IR value at T1) for children with weight loss between the surveys and highest (with a mean delta z of 0.64) for children with substantial weight gain between T0 and T1. Both, children with a low BMI (thin or normal weight) at both measurements and children with a high BMI (overweight or obese) in both surveys, also showed increased values of HOMA-IR in T1, with a higher increase for the overweight or obese children.

**Table 3:** Two-year change of HOMA-IR by changes of weight status

BMI^a^ at baseline	BMI^a^ at follow-up	N	Two-year change of HOMA-IR delta z-IR^b^ (Std^c^)
Overweight/obese	Thin/normal weight	75	−0.19 (1.1)
Thin/normal weight	Thin/normal weight	2539	0.20 (1.2)
Overweight/obese	Overweight/obese	513	0.46 (1.1)
Thin/normal weight	Overweight/obese	221	0.64 (1.2)
All		3348	0.26 (1.2)

^a^weight status according to extended IOTF criteria (Cole, 2012)

^b^delta z-IR = z-score IR (T1) - z-score IR(T0)

^c^standard deviation

Possible baseline determinants of IR at follow-up were analysed in univariate logistic regression models (Table 4). Crude ORs are shown for all children and for normal weight children only. IR at T1 was positively associated with female sex, increasing age, low SES (maximum parental ISCED of three or lower), overweight and obesity, waist circumference, sleep duration ≤ 9 h/night, media consumption (more than 7 h/week of audio-visual media time) and number of audio-visual media in bedroom (any media). No associations were observed for the consumption of sugar or fat. MVPA at baseline (upper two quartiles) showed a protective effect on the development of IR 2 years later. This remained unchanged when the analysis was limited to 5–8 year old children, the age range that the applied cut-off-points by Evenson [41] were calibrated for (data not shown). When only children with normal weight at baseline were considered, an association with IR at follow-up was still evident for sex, low SES, media consumption and audio-visual media in bedroom and MVPA (only 3^rd^ quartile). We also looked at different anthropometric markers (skinfolds, fat free mass from BIA, weight-to-height-ratio, waist-to-height-ratio) but there were only little differences in the associations of these markers with HOMA-IR (data not shown) and the strongest associations were seen for BMI and waist circumference.

**Table 4:** Determinants of IR (HOMA-IR ≥p95^a^) from univariate logistic regression analyses (children without IR at T0)

	Children of all weight groups				Normal weight children only			
	N	%	OR	95 % CI	N	%	OR	95 % CI
Sex	**3152 (all)**				**2297 (all)**			
Female	1508	47.8	**1.5**	**1.3–1.9**	1078	46.9	**1.4**	**1.1–1.9**
Male	1644	52.2	Ref.		1219	53.1	Ref.	
Age (continuous, unit change from mean)	**3152 (all)**		**1.2**	**1.1–1.3**	**2297 (all)**		**1.1**	**1.0–1.2**
ISCED	**3114 (all)**				**2269 (all)**			
Low (1–2)	215	6.9	**2.6**	**1.9–3.6**	135	6.0	**2.2**	**1.4–3.5**
Medium (3–4)	1563	50.2	**1.8**	**1.4–2.3**	1121	49.4	**1.6**	**1.2–2.2**
High (5–6)	1336	52.9	Ref.		1013	44.7	Ref.	
BMI (Cole)	**3152 (all)**							
Thin	362	11.5	**0.5**	**0.3–0.9**				
Normal weight	2297	72.9	Ref.					
Overweight	345	11.0	**4.1**	**3.3–5.0**				
Obese	148	4.7	**10.8**	**8.2–14.0**				
BMI z-score (Cole)	**3152 (all)**							
1^st^ Quartile (≤ −0.49)	813	26.2	0.7	0.5–1.1				
2^nd^ Quartile (−0.49 - ≤0.19)	802	25.6	Ref.					
3^rd^ Quartile (0.19 - ≤0.97)	811	23.1	1.2	0.9–1.8				
4^th^ Quartile (≥ 0.97)	726	25.1	**4.9**	**3.7–6.6**				
BMI z-score (Cole) (unit change from mean)	**3152 (all)**		**2.5**	**2.2–2.8**				
Waist z-score (Cole)	**3152 (all)**							
1^st^ Quartile (≤ −0.66)	826	26.2	0.8	0.5–1.2				
2^nd^ Quartile (−0.66 - ≤0.09)	791	25.1	Ref.					
3^rd^ Quartile (0.09 - ≤0.95)	808	25.6	**1.5**	**1.0–2.1**				
4^th^ Quartile (≥ 0.95)	727	23.1	**5.7**	**4.1–7.7**				
Waist z-score (Cole) (unit change from mean)	**3152 (all)**		**2.2**	**2.0–2.4**				
Audio-visual media time	**3152 (all)**				**2297 (all)**			
≤1 h/day	1062	33.7	Ref.		774	33.7	Ref.	
1 - ≤2 h/day	1221	38.7	**1.4**	**1.1–1.9**	899	39.1	**1.4**	**1.0–2.1**
2 - ≤3 h/day	603	19.1	**1.7**	**1.3–2.3**	439	19.1	**1.8**	**1.2–2.6**
>3 h/day	266	8.4	**2.1**	**1.5–2.9**	185	8.1	**2.3**	**1.5–3.6**
Media in bedroom	**3090 (all)**				**2250 (all)**			
0 media	1902	61.5	Ref.		1429	63.5	Ref.	
1–2 media	868	28.1	**1.8**	**1.4–2.2**	609	27.1	**1.8**	**1.3–2.3**
3 media	320	10.4	**2.6**	**2.0–3.4**	212	9.4	**2.4**	**1.7–3.4**
Sugar consumption propensity score (*N* = 3125)^c^	**3152 (all)**				**2297 (all)**			
1^st^ Quartile (≤ 16.9)	842	26.7	Ref.		625	27.2	Ref.	
2^nd^ Quartile (16.9 - ≤24.1)	832	26.4	1.0	0.8–1.4	557	24.3	1.0	0.7–1.5
3^rd^ Quartile (24.1 - ≤32.4)	766	24.3	1.1	0.8–1.4	562	24.5	1.0	0.7–1.5
4^th^ Quartile (≥ 32.4)	713	22.6	1.2	0.9–1.6	553	24.1	1.4	1.0–1.9
Fat consumption propensity score (*N* = 3125) ^c^	**3152 (all)**				**2297 (all)**			
1^st^ Quartile (≤ 19.0)	901	28.6	Ref.		595	25.9	Ref.	
2^nd^ Quartile (19.0 - ≤24.8)	790	25.1	0.8	0.6–1.0	543	23.6	1.1	0.8–1.6
3^rd^ Quartile (24.8 - ≤31.3)	748	23.7	0.8	0.6–1.0	574	25.0	1.1	0.8–1.6
4^th^ Quartile (≥ 31.3)	713	22.6	0.9	0.7–1.2	585	25.5	1.1	0.8–1.6
Sleep duration on weekdays (*N* = 1730)	**1730 (all)**				**1253 (all)**			
Average sleep time <9 h	104	6.0	**1.8**	**1.2–2.7**	58	4.6	1.5	0.8–2.8
Average sleep time ≥9 h	1626	94.0	Ref.		1195	95.4	Ref.	
Time spent in MVPA^b^	**1042 (all)**				**771 (all)**			
1^st^ Quartile (≤ 27 min./day)	259	24.9	Ref.		203	26.3	Ref.	
2^nd^ Quartile (27 - ≤38.7 min./day)	256	24.6	0.8	0.5–1.2	193	25.0	0.9	0.5–1.4
3^rd^ Quartile (38.7 - ≤54.6 min./day)	264	25.3	**0.4**	**0.2–0.7**	193	25.0	**0.4**	**0.2–0.9**
4^th^ Quartile (≥ 54.6 min./day)	263	25.2	**0.6**	**0.4–1.0**	182	23.6	0.7	0.3–1.0

Figures in **bold** indicate a **significant** effect. Confidence bounds of 1.0 are caused by rounding

^a^Age- and sex-specific 95^th^ percentiles (p95) from Peplies et al. [29]

^b^ *MVPA* moderate to vigorous physical activity

^c^Propensity to consume items high in sugar or fat resp., relative to frequency of all items on food frequency questionnaire

Results from multiple logistic regression analysis are presented in Table 5. Sex (being female), BMI (z-score) and waist circumference (z-score) at baseline were the strongest predictors of HOMA-IR at T1. MVPA showed the same pattern of association as in the univariate analysis: results were strongest for the 3^rd^ quartile. A small but significant increase of risk was also seen for audio-visual media time and fat consumption score. ISCED, age and country showed no influence on HOMA-IR in the multivariate model.

**Table 5:** Determinants of IR (HOMA-IR ≥p95^a^) - results from multivariate mixed logistic regression models

*N* = 1083	Model 1^b^ (with BMI z-score)		Model 2^b^ (with waist z-score)	
	OR	95 % CI	OR	95 % CI
BMI z-score (Cole)				
(unit change from mean)	**2.6**	**2.1–3.1**		
Waist z-score (Cole)				
(unit change from mean)			**2.2**	**1.9–2.6**
Time spent in MVPA^c^				
1^st^ Quartile (≤ 27 min./day)	Ref.		Ref.	
2^nd^ Quartile (27 - ≤38.7 min./day)	0.9	0.6–1.5	1.1	0.7–1.7
3^rd^ Quartile (38.7 - ≤54.6 min./day)	**0.5**	**0.3–0.9**	**0.5**	**0.3–0.9**
4^th^ Quartile (≥ 54.6 min./day)	0.7	0.5–1.1	0.7	0.5–1.1
Sex				
(female versus male)	**2.2**	**1.5–3.1**	**2.5**	**1.8–3.6**
Age				
(unit change from mean)	1.0	0.9–1.2	1.0	0.9–1.1
ISCED				
Low (1–2)	1.2	0.7–2.2	1.3	0.7–2.3
Medium (3–4)	1.2	0.9–1.8	1.2	0.8–1.8
High (5–6)	Ref.	Ref.	Ref.	Ref.
Audio-visual media time (h/d)				
(unit change from mean)	**1.2**	**1.0–1.4**	**1.2**	**1.0–1.4**
Fat consumption propensity score^d^				
(unit change from mean)	**1.2**	**1.0–1.4**	**1.2**	**1.0–1.4**

Figures in **bold** indicate a **significant** effect. Confidence bounds of 1.0 are caused by rounding

^a^Age- and sex-specific 95^th^ percentiles (p95) from Peplies et al. [29]

^b^Analyses were adjusted for all parameters in the respective column, including country as a random effect to account for the clustered study design

^c^ *MVPA* moderate to vigorous physical activity

^d^Propensity to consume items high in or fat, relative to frequency of all items on food frequency questionnaire. The score was included into the model in a modified form: it was divided by 10 to obtain meaningful effect estimates – one unit in the multivariate model thus represents 10 units of the original score used in the univariate model

## Discussion

This study shows a strong prospective association between weight status and HOMA-IR in preadolescent European children and a protective effect of MVPA. It indicates that a longitudinal reduction of BMI also leads to a decline in HOMA-IR and thus a favourable change in metabolic status. The study describes country-specific prevalence rates of IR in the IDEFICS cohort, showing an increasing trend of IR prevalence with BMI category.

Prevalence proportions of IR found in our study (10.9 % in normal weight, 26.5 % in overweight, 66.7 % in obese children) were in agreement with those reported in the literature, even though comparability is limited, because previous studies were based on older children and used different definitions of IR. A Chilean cohort of 10–15 year old children displayed prevalence values of 13 % in normal weight, 37.1 % in overweight, and 61.6 % in obese children, using HOMA-IR ≥ p90 as cut point for IR [21]. An US-American survey on 12–19 year old adolescents [8] used a cut point of HOMA-IR ≥p97.5, which resulted in prevalence values of about 4 % in normal weight, 16 % in overweight and 52 % in obese US-adolescents aged 12–19 years (data taken from figure). As it was previously shown that insulin levels and thus also HOMA-IR levels show a peak round the age of 13 to 14 years [44], slightly lower prevalence proportions for older adolescents are plausible.

In our study, weight status and waist circumference at baseline appeared as the main risk factors for IR at follow-up, but sex and lifestyle indices (objectively determined PA, fat consumption score, audio-visual media time, and media in bedroom) were also associated with incidence of IR. The age effect which was seen in the univariate analysis can probably be attributed in large part to the increase of overweight and obesity with age. The higher risk of IR in girls compared to boys also persisted when older children (>7 years at baseline) were excluded from the analysis to avoid possible influences of early puberty. Fat consumption, as expressed by a propensity score [38, 39], was also connected with IR risk in the multivariate model. The PA level of children in our study was rather low, and only a small proportion of children (24.2 % of the boys and 9.7 % of the girls) reached the daily activity level recommended by the World Health Organization (≥ 60 min/ day) [45]. Considering this and the fact that accelerometer data were only available for about one third of the study population, the protective effect on IR found for MVPA is certainly noteworthy, despite the lack of a clear trend.

The associations observed in this study confirm previous findings, which however are mainly based on studies in older children/ adolescents and on cross-sectional data. A Spanish cross-sectional study on cardiovascular risk factors in 6 to 8 year-old schoolchildren found some of the same metabolic consequences of obesity as in adults (elevated triglycerides, insulin, HOMA-IR, and lower HDL-cholesterol) [46]. In a representative subsample of diabetes-free US-adolescents aged 12–19 years who participated in the National Health and Nutrition Examination Survey (NHANES), obesity was by far the most important determinant of IR, independent of sex, age, or race/ethnicity, but data on PA was not included in the analysis [8]. Subcutaneous adiposity was also the most significant covariate for HOMA-IR in a family-based US-American study, including children and adolescents from 6 years on [9]. A large insulin screening study conducted on 10–14 year old children in a methodological context, using a blood spot assay on filter paper, indicated associations of pubertal stage and measures of central and peripheral adiposity with insulin level [10]. In the IDEFICS baseline survey, a low amount of physical activity was shown to be associated with a cluster of CVD risk factors including IR [13]. In accordance with the evidence from our analysis, a few other studies published data on the association of dietary factors like intake of total energy, total fat or saturated fat with IR in children and adolescents [11, 12].

When longitudinal change of HOMA-IR (delta z-IR) was linked to overweight and obesity, children who retained their low or normal weight until the follow-up survey showed a moderate increase of their IR z-score, while children who were overweight or obese in both surveys showed a stronger increase of their IR z-score. In both cases, the mean BMI increased within the defined weight groups. In the small group of children (*N* = 75) who had changed to a lower weight group at follow-up, the delta z-IR values also declined. The highest gain in IR was seen for children who changed to a higher weight group.

There is a solid body of evidence for the association of obesity and physical inactivity with IR, especially in adults, but there is a controversy on whether the influence of sedentary lifestyle on IR is mediated by obesity, whether both are independent predictors of this condition, or whether the risk for IR involved with obesity is modified by PA. In our study, the association of MVPA with IR is attenuated only slightly when only children with normal weight at baseline are considered, i.e. MVPA reduces the risk of developing IR, also for children with normal weight at baseline which indicates that the effect of missing physical activity is not just mediated by obesity. This is also confirmed by the results of the multivariate model.

Other studies have also shown that increased PA can reduce the risk of cardiovascular disease and metabolic risk factors including insulin, already in children [47–49]. Obesity might thus be the main determinant of IR, but its effect can be attenuated by a sufficient amount of PA. An US-American cross-sectional study [50] included 8–17 year olds with a BMI ≥85th percentile who were enrolled in a multidisciplinary paediatric weight management clinic. MVPA was the strongest independent predictor of metabolic health in these overweight and obese children. A review [51] on the therapeutic power of PA in children suggests that PA may have greater influence on body composition and cardiovascular risk factors than dieting as it possibly modulates the fuel metabolism. The increased fat oxidation by PA might be the basis for prevention and restoration of insulin sensitivity and reduction of MS in obese children.

In an analysis on NHANES data from obese adolescents and adults [52], self-reported PA was associated with a metabolically healthy phenotype in adults, but not in adolescents. A large cross-sectional study among Spanish adults [23] provided evidence that PA is one of the main factors responsible for a healthy phenotype among the obese. Only few studies published results for adolescents and these showed no association between PA and metabolically healthy obesity [22, 52]. Further research in children is needed, as there are no studies on metabolically healthy obesity in children. A cross-sectional analysis of IDEFICS baseline data showed the importance of PA to protect against clustering of CVD risk factors [13]. In preschool and even more so in school children (≤9 years), CVD risk was elevated for children in the lower quintiles of MVPA.

The main strengths of this study are the large study size, its longitudinal design, the highly standardised data collection across different European countries, and the young age of the examined children, as well as the fact that PA was measured objectively by activity monitors. There are on the other hand also some limitations that should be mentioned: Pubertal stage was not assessed in the IDEFICS surveys and it can be suspected that a considerable proportion of the older children might have already started into puberty at follow-up. During the German Health Interview and Examination Survey for Children and Adolescents (KiGGS) [53], at an age of 10 years, 42.4 % of girls and 35.7 % of boys reported the development of pubic hair. Early puberty is thus more likely in girls and especially in girls, insulin concentrations have been shown to have a distinct pubertal peak [44]. Nevertheless, as mentioned above, the elevated IR risk seen in girls remained unchanged when older children (>7 years) where excluded. Food consumption scores used in this study were based on a food frequency questionnaire which neither included school meals nor portion sizes and were thus calculated from average quantities which might reduce the truly existing differences. Presented prevalence rates only apply for the study population at hand. These should neither be transferred to the respective countries in general nor to other study populations.

## Conclusions

This study is, to our knowledge, the first study to show longitudinal data on IR in a preadolescent children’s population. It supports the available evidence, that overweight and obesity are the main determinants of IR, while PA seems to ameliorate the risk, independent of weight status. Reduction of weight is thus an important measure in the fight against IR in children, but children should above all be encouraged to engage in regular PA, as this will keep them metabolically healthy even under the presence of overweight/obesity.

## Abbreviations

BIA: Bioelectrical impedance analysis
BMI: Body mass index
HOMA-IR: Homeostasis model assessment to quantify insulin resistance
IDEFICS (acronym): Identification and prevention of dietary- and lifestyle-induced health effects in children and infants
IR: Insulin resistance
ISCED: International Standard Classification of Education
MS: Metabolic syndrome
MVPA: Time spent in moderate to vigorous physical activity
OR: Odds ratio
PA: Physical activity
SACINA: Self-administered children and infants nutrition assessment (a standardized 24-h recall method)
